# A Developmental Switch in the Response of DRG Neurons to ETS Transcription Factor Signaling

**DOI:** 10.1371/journal.pbio.0030159

**Published:** 2005-04-26

**Authors:** Simon Hippenmeyer, Eline Vrieseling, Markus Sigrist, Thomas Portmann, Celia Laengle, David R Ladle, Silvia Arber

**Affiliations:** **1**Biozentrum, Department of Cell BiologyUniversity of Basel, Switzerland and Friedrich Miescher Institute, BaselSwitzerland; Harvard UniversityUnited States of America

## Abstract

Two ETS transcription factors of the Pea3 subfamily are induced in subpopulations of dorsal root ganglion (DRG) sensory and spinal motor neurons by target-derived factors. Their expression controls late aspects of neuronal differentiation such as target invasion and branching. Here, we show that the late onset of ETS gene expression is an essential requirement for normal sensory neuron differentiation. We provide genetic evidence in the mouse that precocious ETS expression in DRG sensory neurons perturbs axonal projections, the acquisition of terminal differentiation markers, and their dependence on neurotrophic support. Together, our findings indicate that DRG sensory neurons exhibit a temporal developmental switch that can be revealed by distinct responses to ETS transcription factor signaling at sequential steps of neuronal maturation.

## Abstract 2

By expressing ETS transcription factors at different developmental stages of dorsal root ganglion development, the authors show that late onset of ETS expression is essential for normal sensory neuron differentiation

## Introduction

Neuronal differentiation is a protracted process during which newly generated neurons express distinct cellular and molecular programs at precise times during their maturation: long-distance axon outgrowth, subsequent terminal branching, and finally synaptogenesis. Many important aspects of neuronal character appear to be acquired through the expression of transcription factors at progenitor cell stages, whereas others depend on expression immediately upon cell cycle exit [1]. But whether the orderly expression and activity of transcriptional programs at much later developmental stages, well after cell cycle exit, is an essential step in the progression of neuronal differentiation and circuit assembly has yet to be resolved.

The differentiation of sensory neurons of dorsal root ganglia (DRG) has been studied extensively with respect to inductive events that specify neuronal fate [2,3], as well as the involvement of late target-derived neurotrophic factors in the control of neuronal survival [4]. Recent evidence has begun to emerge that target-derived factors are also involved in regulating later aspects of neuronal differentiation [5,6,7]. In particular, genetic experiments have addressed the survival-independent role of neurotrophic factors during development by exploiting strains of mice defective both in neurotrophin signaling and in the function of the proapoptotic gene *Bax* [8,9]. These studies, for example, have revealed that neurotrophin signaling controls the acquisition of peptidergic traits in nociceptive DRG neurons and the control of target innervation [8,9].

The onset of some transcriptional programs in neurons, however, has also been shown to occur long after neurons exit the cell cycle. An emerging principle from work in *Drosophila* and vertebrates is that target-derived factors play a crucial role in the induction of these transcriptional programs [10]. In *Drosophila,* retrograde BMP signals from the target region control the terminal differentiation of a subpopulation of peptidergic neurons expressing Apterous and Squeeze [11,12]. In vertebrates, peripheral neurotrophic signals have been shown to direct the onset of expression of the ETS transcription factors Er81 and Pea3 in DRG sensory neurons and motor neuron pools several days after these neurons have become post-mitotic [9,13,14,15,16]. Moreover, the induction of Er81 expression in proprioceptive afferents is known to be mediated by peripheral neurotrophin 3 (NT-3) [9]. These two ETS proteins control late aspects of spinal monosynaptic circuit assembly, with Er81 directing proprioceptive sensory neuron differentiation and Pea3 directing motor neuron pool differentiation, respectively [14,15]. In particular, in the absence of *Er81,* achieved by mutation in the gene or by deprivation of peripheral neurotrophin signaling, group Ia proprioceptive afferents fail to invade the ventral spinal cord and to make effective synaptic connections with motor neurons [9,14].

The involvement of target-derived signals in induction of ETS transcription factor expression raises the question of the necessity for the observed delay in the onset of ETS signaling for neuronal maturation. Would precocious expression of ETS proteins in post-mitotic neurons also direct the appropriate sensory neuron developmental programs? In this study, we have used mouse genetics to test this general idea, by investigating whether the precise timing of onset of ETS transcription factor signaling is essential for normal sensory neuron development. We have assessed the biological effects of inducing ETS signaling either at the correct developmental time, or precociously. We find that within proprioceptive sensory neurons, the late onset of ETS signaling is essential for the establishment of normal sensory afferent projections in the spinal cord. Precocious initiation of ETS signaling in post-mitotic DRG neurons leads to abnormal DRG neuron differentiation characterized by neurotrophin-independent neurite outgrowth and inappropriate profiles of gene expression. Our findings reveal that target-triggered inductive signals provide an effective means of ensuring the late onset of expression of transcription factors, and thus an orderly temporal transcriptional sequence that is crucial for neuronal maturation and circuit assembly.

## Results

To test the hypothesis that a temporal delay in the onset of transcriptional programs is crucial for the control of appropriate neuronal maturation, we studied the development of proprioceptive DRG neurons, since transcriptional effectors regulated by target-derived signals, as well as some of their downstream biological actions, have been identified for these neurons. Er81 controls proprioceptive afferent connectivity [14], and we therefore sought to identify an ETS transcriptional regulator that, when expressed over the normal time course of Er81 expression, is able to substitute for Er81 function within group Ia afferent sensory neurons. With this reference point, we then designed experiments to examine the effects of precocious post-mitotic expression of the same ETS transcription factor on sensory neuron differentiation.

### *EWS-Pea3* Can Replace *Er81* Function in Controlling Ia Afferent Projections

**Figure 1:**
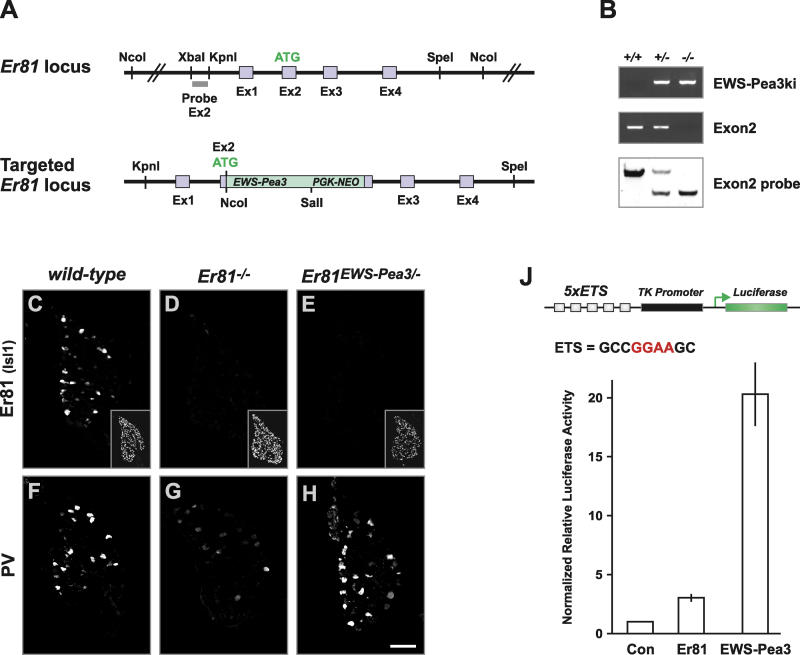
Replacement of *Er81* by *EWS-Pea3* (A) Generation of *Er81^EWS-Pea3^* mutant mice. Above is the organization of the *Er81* genomic locus in the region targeted by homologous recombination in analogy to [14]. Exons 1–4 are shown as light blue boxes, and the *Er81* start codon in exon 2 is indicated as ATG. The probe used to detect homologous recombination is shown as a grey box. Below is replacement of *Er81* by *EWS-Pea3* through the integration of *EWS-Pea3* in frame with the endogenous start codon of the *Er81* locus in exon 2 (in analogy to [14]). (B) PCR and Southern blot analysis of *Er81^EWS-Pea3^* wild-type (*+/+*), heterozygous (*+/−*), and homozygous (*−/−*) genomic DNA to detect the mutant allele. PCR primer pairs (EWS-Pea3ki) were used to detect specifically the recombined allele, and a primer pair in exon2 was used to detect the presence of the wild-type allele [14]. (C–E) Analysis of Er81 expression in lumbar DRG neurons of E16.5 wild-type (C), *Er81^−/−^* (D), and *Er81^EWS-Pea3/−^* (E) embryos. Inset in lower right corner of each panel shows Isl1 expression in the respective DRG. (F–H) PV expression in lumbar DRG of E16.5 wild-type (F), *Er81^−/−^* (G), and *Er81^EWS-Pea3/−^* (H) embryos. Confocal scans were performed with equal gain intensity. (J) Transcriptional transactivation of luciferase expression from a minimal reporter construct containing five consensus ETS DNA-binding sites ( GCCGGAAGC; [18,19]) and a minimal TK promoter upon transient transfection of Er81 (*n* ≥ 7; 3.03 ± 0.66) or EWS-Pea3 (*n* ≥ 7; 20.3 ± 2.7). Relative luciferase activity normalized to control (Con). Scale bar: 80 μm.

We first defined an ETS transcription regulator that is able to replace the function of Er81 within proprioceptive afferents to direct projections into the ventral spinal cord. Er81, Pea3, and Erm constitute the Pea3 subfamily of ETS transcription factors, show a high degree of amino acid identity, and bind to very similar DNA target sequences [17,18,19]. Nevertheless, when introduced into the *Er81* locus in analogy to a previously used targeting strategy (data not shown; [14]), neither *Pea3* nor *Erm* could rescue Ia proprioceptive afferent projections to extensively invade the ventral horn of the spinal cord (data not shown). These findings prompted us to analyze mice in which we integrated *EWS-Pea3,* a break-point fusion product between the amino-terminal domain of the Ewing sarcoma (EWS) gene and the Pea3 DNA binding domain [20,21], into the *Er81* locus (Figure 1). We found that in a luciferase-enzyme-based cell culture transfection assay, EWS-Pea3 showed stronger transactivation activity than Er81 or Pea3 (Figure 1J; data not shown), in agreement with previous studies [22,23,24]. Moreover, transactivation by EWS-Pea3 was abolished by mutation of ETS-binding sites in the reporter plasmid, demonstrating ETS-binding-site dependence (data not shown).

Expression of Er81 in DRG neurons of embryos containing integration of *EWS-Pea3* in the *Er81* locus (*Er81^EWS-Pea3/−^*) was abolished (Figure 1E), and the expression level of the calcium-binding protein Parvalbumin (PV) in proprioceptive afferents, which is decreased approximately 5- to 10-fold in *Er81* mutants [14], was comparable to wild-type levels in *Er81^EWS-Pea3/−^* embryos (Figure 1F–1H). To further define DRG neuron differentiation in the presence of *EWS-Pea3* in proprioceptive afferents in vivo, we assessed whether replacement of *Er81* by *EWS-Pea3* had an influence on neuronal survival or on the expression of proprioceptive-afferent-specific genes. *Er81^EWS-Pea3/−^* mice did not differ from wild-type in the number of proprioceptive afferent cell bodies within the DRG of L1 to L5, the expression of several genes normally expressed by proprioceptive afferents, and the lack of expression of genes not normally expressed in proprioceptive afferents (Figure S1; data not shown). Together, these findings suggest that the expression of EWS-Pea3 from the normal time of onset mimics the function of Er81 as assessed by induction and maintenance of gene expression within proprioceptive afferents.

**Figure 2:**
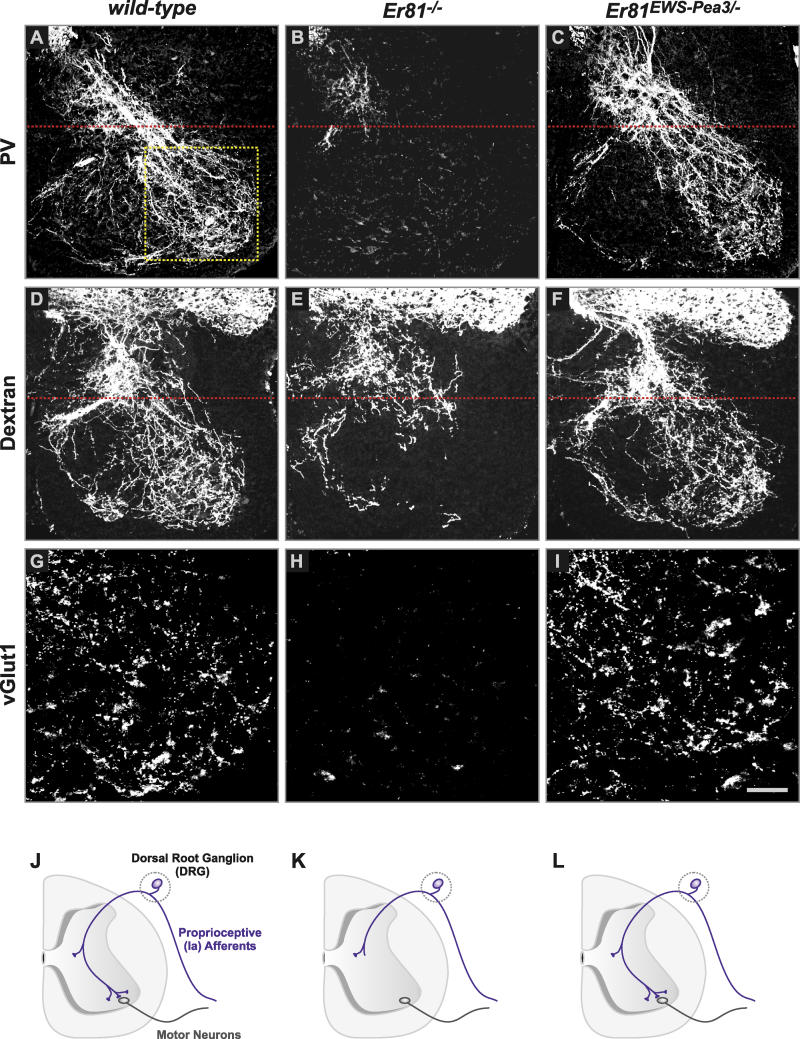
Rescue of Ia Proprioceptive Afferent Projections into the Ventral Spinal Cord in *Er81^EWS-Pea3^* Mutants (A–F) Morphological analysis of central projections at lumbar level L3 of PV^+^ DRG neurons (A–C) or all DRG sensory afferents after application of fluorescently labeled dextran to individual dorsal roots (D–F) in P0.5 (A–C) or P5 (D–F) wild-type (A and D), *Er81^−/−^* (B and E), and *Er81^EWS-Pea3/−^* (C and F) mice. Red dotted line indicates intermediate level of spinal cord. (G–I) Analysis of vGlut1 immunocytochemistry in the ventral horn of P0.5 wild-type (G), *Er81^−/−^* (H), and *Er81^EWS-Pea3/−^* (I) mice. Yellow dotted box in (A) indicates size of images shown in (G–I). (J–L) Schematic summary diagrams of the morphological rescue of Ia proprioceptive afferent projections (blue) into the ventral spinal cord observed in wild-type (J), *Er81^−/−^* (K), and *Er81^EWS-Pea3/−^* (L) mice. DRG indicated by dotted grey line; motor neurons are shown in black. Scale bar: (A–C), 150 μm; (D–F), 160 μm; (G–I), 70 μm.

To determine the extent of rescue of Ia proprioceptive afferent projections into the ventral spinal cord of *Er81* mutant mice achieved by expression of *EWS-Pea3,* we traced intraspinal afferent projections by axonal labeling of PV (Figure 2A–2C). In addition, to analyze axon ingrowth independent of the level of PV expression in DRG neurons, we used anterograde labeling of afferent fibers by applying fluorescently labeled dextran to cut dorsal roots (Figure 2D–2F). Using both assays, we found extensive rescue of the projections into the ventral horn of the spinal cord in *Er81^EWS-Pea3/−^* mice (Figure 2C and 2F). Within the ventral horn, Ia afferents in both wild-type and *Er81^EWS-Pea3/−^* mice formed vGlut1^+^ terminals that were absent in *Er81* mutant mice (Figure 2G–2I). To assess whether synapses between Ia afferents and motor neurons are functional in *Er81^EWS-Pea3/−^* mice, we performed intracellular recordings from identified quadriceps motor neurons after stimulation of nerves innervating the quadriceps muscle group. We found no significant difference in the input amplitude to quadriceps motor neurons when comparing wild-type to *Er81^EWS-Pea3/−^* mice (Figure S2; wild-type, 10.6 ± 0.9 mV, *n* = 11; *Er81^EWS-Pea3/−^*, 10.9 ± 1 mV, *n* = 8). Together, these findings suggest that in the absence of *Er81, EWS-Pea3* can direct the complex biological process of correct laminar termination within the ventral spinal cord and the formation of synapses with motor neurons (Figure 2J–2L), thus identifying an ETS transcription factor suitable for heterochronic expression experiments in DRG neurons.

### Precocious Expression of *EWS-Pea3* in DRG Neurons Leads to Axonal Projection Defects

To address the consequences of precocious ETS signaling for proprioceptive afferent differentiation, we next expressed EWS-Pea3 in DRG neurons as soon as they became post-mitotic. We used a binary mouse genetic system based on Cre-recombinase-mediated excision of a transcriptional stop cassette flanked by *loxP* sites. Targeting cassettes were integrated into the *Tau* locus to generate two strains of mice conditionally expressing either *EWS-Pea3* or a membrane-targeted green fluorescent protein (mGFP) to trace axonal projections of DRG neurons (Figure S3; [25]). Embryos positive for either *Isl1^Cre/+^* and *Tau^EWS-Pea3/+^* or *Isl1^Cre/+^* and *Tau^mGFP/+^* alleles showed efficient activation of the silent *Tau* allele in 95% or more of all DRG neurons, including proprioceptive afferents, at all segmental levels (Figure S3).

**Figure 3:**
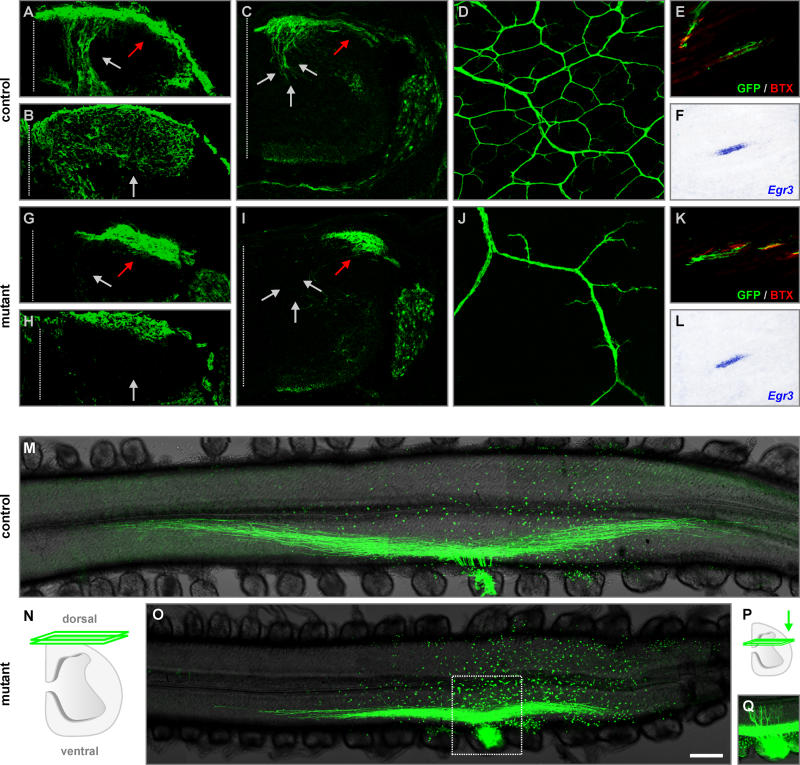
Defects in the Establishment of Sensory Afferent Projections upon Precocious Expression of *EWS-Pea3* in DRG Neurons (A–C and G–I) Visualization of sensory afferent projections (green) into the spinal cord of wild-type (A–C) and *Tau^EWS-Pea3/+^ Isl1^Cre/+^* (G–I) embryos at E13.5 (A, C, G, and I) and E16.5 (B and H) by Cre-recombinase-mediated activation of mGFP expression from the *Tau* locus (A, B, G, and H) or by a *Thy1^spGFP^* transgene (C and I; [25]). Grey arrows indicate normal pattern of afferent projections into the spinal cord, whereas red arrows show aberrant accumulation of sensory afferents at the lateral edge of the spinal cord in *Tau^EWS-Pea3/+^ Isl1^Cre/+^* embryos. (D–F and J–L) Analysis of sensory afferent projections (green) into the skin (D and J) or muscle (E and K; red, α-Bungarotoxin, BTX) of wild-type (D–F) and *Tau^EWS-Pea3/+^ Isl1^Cre/+^* (J–L) embryos at E16.5 by Cre-recombinase-mediated activation of mGFP (D, E, J, and K) expression from the *Tau* locus. (F and L) show *Egr3* expression in intrafusal muscle fibers using in situ hybridization (consecutive sections to [E and K] are shown). (M–Q) Analysis of bifurcation of sensory afferent projections towards the spinal cord in E13.5 wild-type (M) and *Tau^EWS-Pea3/+^ Isl1^Cre/+^* (O and Q) embryos after injection of fluorescently labeled dextran (green) into one DRG (lumbar level L3). Confocal scanning plane for (M and O) is schematically illustrated in (N). Inset in (O) is also shown at a deeper confocal scanning plane (P and Q) to visualize aberrant axonal projections. Scale bar: (A and G), 60 μm; (B and H), 80 μm; (C and I), 100 μm; (D and J), 160 μm; (E, F, K, and L), 70 μm; (M, O, and Q), 240 μm.

We first assessed the influence of *EWS-Pea3* expression in early post-mitotic DRG neurons on the establishment of afferent projections into the spinal cord using the *Tau^mGFP/+^* allele or a *Thy1*-promoter-driven synaptophysin green fluorescent protein (spGFP) with an expression profile restricted to DRG sensory neurons at embryonic day (E) 13.5 (*Thy1^spGFP^;* [25]) (Figure 3). In contrast to wild-type proprioceptive afferent projections (Figure 3A–3C), GFP^+^ sensory afferents in *Tau^EWS-Pea3/+^ Isl1^Cre/+^* embryos failed to invade the spinal cord and instead were found in an extreme lateral position at the dorsal root entry zone, a phenotype observed at least up to E18.5 (Figure 3A–3C and 3G–3I; data not shown). We next visualized the path of sensory afferent projections towards the dorsal root entry zone in *Tau^EWS-Pea3/+^ Isl1^Cre/+^* embryos by injecting fluorescently labeled dextran into an individual DRG (L3; *n* = 3; Figure 3M–3Q). Sensory afferents in E13.5 wild-type embryos bifurcated at their lateral spinal entry point, and projected rostrally and caudally over six or more segmental levels while gradually approaching the midline (Figure 3M). Sensory afferents in *Tau^EWS-Pea3/+^ Isl1^Cre/+^* embryos also bifurcated at the entry point, although approximately 5% of afferent fibers continued to grow towards the midline (Figure 3O and 3Q). While rostro-caudal projections were present in *Tau^EWS-Pea3/+^ Isl1^Cre/+^* embryos, afferent fibers failed to approach the midline at distal segments and continued to occupy an extreme lateral position (Figure 3O), consistent with the analysis of transverse sections.

We next examined the establishment of peripheral projections upon precocious *EWS-Pea3* expression in DRG neurons. While sensory axons in *Tau^EWS-Pea3/+^ Isl1^Cre/+^* embryos reached the skin and established major nerve trunks by E16.5, only rudimentary sensory axon branching was established within the skin (Figure 3D and 3J). In addition, there was a significant reduction in the number of muscle spindles in *Tau^EWS-Pea3/+^ Isl1^Cre/+^* embryos (approximately 25% of wild-type complement; *n* = 3) as assessed by innervation and expression of genes specific for intrafusal muscle fibers such as *Egr3* (Figure 3E, 3F, 3K, and 3L; [26]). In summary, whereas isochronic expression of *EWS-Pea3* promoted the establishment of proprioceptive afferent projections into the ventral spinal cord, precocious expression of the same ETS signaling factor in DRG neurons interfered with establishment of projections into the spinal cord as well as to peripheral targets.

### Precocious *EWS-Pea3* Expression Promotes Neurotrophin-Independent Survival and Neurite Outgrowth

To begin to address the cellular and molecular mechanisms involved in the distinct biological actions of *EWS-Pea3* at different developmental stages, we first turned to in vitro culture experiments. These experiments permit assessment of whether precocious ETS transcription factor signaling influences neuronal survival and in vitro neurite outgrowth of DRG neurons, two parameters prominently influenced by target-derived neurotrophic factors and their receptors.

**Figure 4:**
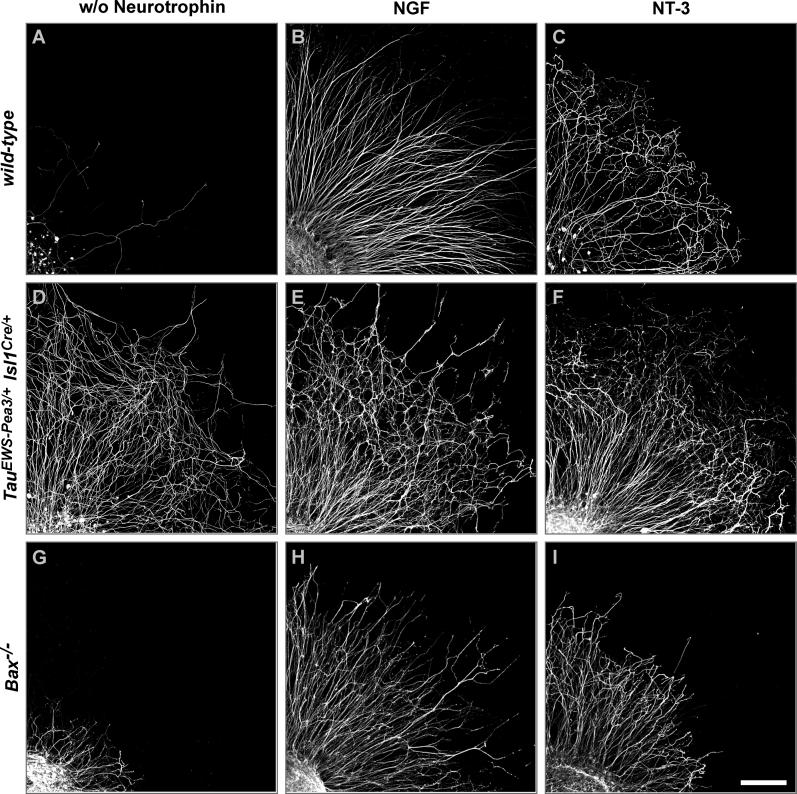
Neurotrophin-Independent Neurite Outgrowth In Vitro of DRG Neurons Expressing EWS-Pea3 Precociously E13.5 lumbar DRG from wild-type (A, B, and C), *Tau^EWS-Pea3/+^ Isl1^Cre/+^* (D, E, and F), or *Bax^−/−^* (G, H, and I) embryos cultured for 48 h without neurotrophic support (A, D, and G) or in the presence of NGF (B, E, and H) or NT-3 (C, F, and I) were stained for expression of neurofilament to visualize axonal extensions. Scale bar: 130 μm.

We cultured E13.5 whole DRG explants from wild-type and *Tau^EWS-Pea3/+^ Isl1^Cre/+^* embryos in the presence of NGF or NT-3 or in the absence of neurotrophins and analyzed neuronal survival and neurite outgrowth on matrigel substrate after 48 h in vitro. Without neurotrophic support, very few wild-type DRG neurons survived (Figure 4A). In contrast, culturing wild-type DRG with neurotrophic factors led to neuronal survival and neurite outgrowth. Addition of NGF, which supports survival of cutaneous afferents, resulted in straight and unbranched neurite outgrowth (Figure 4B), while cultures grown in the presence of NT-3, which supports survival of proprioceptive afferents, resulted in a highly branched neurite outgrowth pattern after 48 h in vitro (Figure 4C). Surprisingly, DRG neurons isolated from *Tau^EWS-Pea3/+^ Isl1^Cre/+^* embryos and cultured without neurotrophic support survived after 48 h in vitro and had established long and highly branched neurites (Figure 4D). Neither the pattern of neurite outgrowth nor neuronal survival changed significantly after application of either NGF or NT-3 (Figure 4E and 4F).

**Figure 5:**
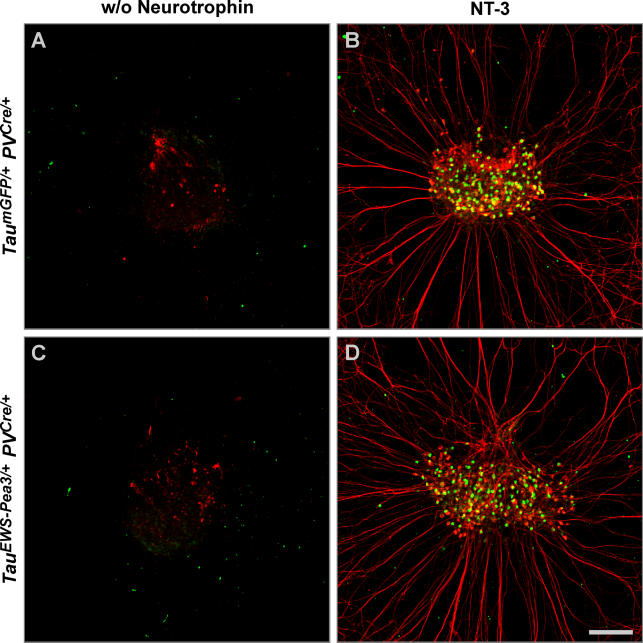
DRG Neurons Expressing EWS-Pea3 Isochronically Depend on Neurotrophins for Survival E14.5 lumbar DRG from *Tau^mGFP/+^ PV^Cre/+^* (A and B) and *Tau^EWS-Pea3/+^ PV^Cre/+^* (C and D) embryos cultured for 48 h without neurotrophic support (A and C) or in the presence of NT-3 (B and D) were stained for expression of neurofilament (red) and LacZ (green) to visualize axonal extensions and survival of PV-expressing proprioceptive afferents. Scale bar: 150 μm.

To directly compare neurotrophin dependence of DRG neurons expressing *EWS-Pea3* from the *Tau* locus at a precocious versus isochronic time of onset, we generated a strain of mice in which *Cre* recombinase is expressed from the *PV* locus (Figure S4). The expression of GFP in *Tau^mGFP/+^ PV^Cre/+^* was restricted to PV^+^ proprioceptive DRG neurons and mirrored the onset of expression of PV at approximately E14 (Figure S4; data not shown). We next cultured E14.5 whole DRG explants from *Tau^EWS-Pea3/+^ PV^Cre/+^* and *Tau^mGFP/+^ PV^Cre/+^* mice for 48 h in vitro in the presence or absence of NT-3 (Figure 5). We found that DRG neurons from both genotypes survived and extended neurites only in the presence of NT-3, whereas they died in the absence of NT-3 (Figure 5). Together, these findings suggest that only precocious but not isochronic ETS signaling in DRG neurons is capable of uncoupling survival and neurite outgrowth from a requirement for neurotrophin signaling normally observed in wild-type DRG.

To determine whether neuronal survival of DRG neurons from *Tau^EWS-Pea3/+^ Isl1^Cre/+^* embryos in the absence of neurotrophic support is sufficient to explain the observed neuronal outgrowth, we analyzed DRG isolated from mice mutant in the proapoptotic gene *Bax* [27]. Consistent with previous results, *Bax^−/−^* DRG neurons survived without neurotrophic support [28]. In contrast, neurite outgrowth of *Bax^−/−^* DRG neurons was significantly less (see Figure 4G) than that of either DRG from *Tau^EWS-Pea3/+^ Isl1^Cre/+^* embryos cultured in the absence of neurotrophic support (see Figure 4D) or *Bax^−/−^* DRG neurons cultured in the presence of neurotrophic support (see Figure 4H and 4I). These findings suggest that in addition to mediating neurotrophin-independent neuronal survival, expression of *EWS-Pea3* in early post-mitotic neurons also promotes neurite outgrowth in a neurotrophin-independent manner.

**Figure 6:**
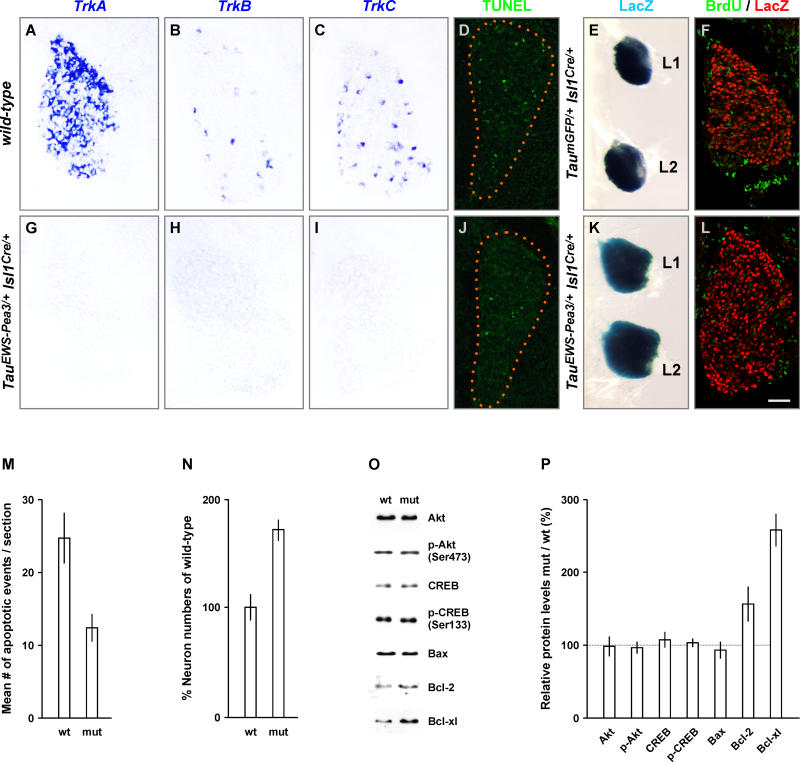
Loss of Trk Receptor Expression and Increased Survival in DRG Neurons upon Precocious ETS Signaling (A–C and G–I) In situ hybridization analysis of *TrkA* (A and G), *TrkB* (B and H), and *TrkC* (C and I) expression in E16.5 lumbar DRG of wild-type (A–C) and *Tau^EWS-Pea3/+^ Isl1^Cre/+^* (G–I) embryos. (D–F and J–L) Analysis of lumbar DRG of wild-type (D), *Tau^mGFP/+^ Isl^Cre/+^* (E and F), and *Tau^EWS-Pea3/+^ Isl1^Cre/+^* (J, K, and L) embryos for (1) neuronal cell death at E13.5 by TUNEL (green; D and J), (2) cell survival and proliferation at E16.5 by LacZ (blue) wholemount staining (E and K; lumbar levels L1 and L2 are shown), and (3) BrdU (green)/LacZ (red) double labeling (F and L). (M and N) Quantitative analysis (*n* ≥ 3 independent experiments) of the mean number of apoptotic events relative to wild-type levels is shown in (M) and neuronal survival in (N) as percent of wild-type of DRG at lumbar levels L1 to L5 as quantified on serial sections. (O) Western blot analysis of protein extracts isolated from lumbar DRG of E16.5 wild-type (wt) and *Tau^EWS-Pea3/+^ Isl1^Cre/+^* (mut) embryos using the following antibodies: Akt, p-Akt (Ser473), CREB, p-CREB (Ser133), Bax, Bcl2, and Bcl-xl. (P) Quantitative analysis of protein levels relative to wild-type in percent is shown on the right (*n* = 3 independent experiments). Scale bar: (A–C and G–I), 35 μm; (D and J), 40 μm; (E and K), 200 μm; (F and L), 50 μm.

To begin to assess at which step of the neurotrophin signaling cascade DRG neurons from *Tau^EWS-Pea3/+^ Isl1^Cre/+^* embryos become unresponsive to the addition of neurotrophins, we assayed the expression of neurotrophin receptors in *Tau^EWS-Pea3/+^ Isl1^Cre/+^* embryos (Figure 6). Whereas expression of the neurotrophin receptors *TrkA*, *TrkB,* and *TrkC* marks afferents of distinct sensory modalities in DRG of wild-type embryos (Figure 6A–6C) [4,29], *Tau^EWS-Pea3/+^ Isl1^Cre/+^* embryos showed complete absence of expression of *TrkA*, *TrkB,* and *TrkC* in DRG neurons at E16.5 (Figure 6G–6I). This absence of Trk receptor expression in DRG of *Tau^EWS-Pea3/+^ Isl1^Cre/+^* embryos provides a likely explanation for the lack of responsiveness of these neurons to the addition of neurotrophic factors.

We next assayed whether the complete absence of *Trk* receptor expression in *Tau^EWS-Pea3/+^ Isl1^Cre/+^* embryos had an influence on naturally occurring cell death in vivo using TUNEL on DRG sections. Surprisingly, we found that apoptosis was decreased by approximately 50% (*n* = 3 embryos, average of >50 sections) in DRG of *Tau^EWS-Pea3/+^ Isl1^Cre/+^* embryos in comparison to wild-type (Figure 6D, 6J, and 6M). Quantitative analysis of the number of neurons in lumbar DRG of *Tau^EWS-Pea3/+^ Isl1^Cre/+^* embryos revealed a significant increase to approximately 170% of wild-type levels (Figure 6E, 6K, and 6N). Moreover, BrdU pulse-chase experiments ruled out the possibility that DRG neurons in *Tau^EWS-Pea3/+^ Isl1^Cre/+^* embryos reenter the cell cycle (no BrdU^+^/LacZ^+^ cells, *n* = 3 embryos, analysis of >50 sections each; Figure 6F and 6L). Together with the in vitro culture experiments, these findings suggest that DRG neurons from *Tau^EWS-Pea3/+^ Isl1^Cre/+^* embryos remain post-mitotic but fail to become sensitive to naturally occurring cell death, and survive in the absence of *Trk* receptors and neurotrophic support.

We next analyzed whether changes in the expression of proteins known to be involved in the regulation of neuronal survival or cell death could be detected in DRG of *Tau^EWS-Pea3/+^ Isl1^Cre/+^* embryos. We found no significant quantitative changes in the level of Akt/p-Akt or CREB/p-CREB in DRG (Figure 6O and 6P) both of which have been shown to be key regulators of neuronal survival [29]. Moreover, the level of the proapoptotic Bcl2 family member Bax was not significantly reduced (Figure 6O and 6P). In contrast, the expression level of the anti-apoptotic Bcl2 family members Bcl-xl and Bcl2 was significantly increased when compared to wild-type levels (Bcl2, 157%; Bcl-xl, 259%; average of *n* = 3 independent experiments; Figure 6O and 6P), providing a potential molecular explanation for the enhanced neuronal survival of DRG neurons of *Tau^EWS-Pea3/+^ Isl1^Cre/+^* embryos in the absence of *Trk* receptor expression [30].

### Only Precocious but Not Isochronic ETS Signaling in DRG Neurons Interferes with Neuronal Fate Acquisition

The observed differences in neuronal survival and neurite outgrowth between precocious and isochronic expression of EWS-Pea3 prompted us to perform a direct comparative analysis of gene expression between mice with precocious EWS-Pea3 expression and mice in which the expression of *EWS-Pea3* is initiated in DRG sensory neurons from the time of normal onset of Er81 expression. Moreover, to rule out the possibility that a differential effect may be due to the different genetic strategies by which expression of *EWS-Pea3* in proprioceptive afferents is achieved, we performed this analysis both in *Er81^EWS-Pea3/−^* and *Tau^EWS-Pea3/+^ PV^Cre/+^* embryos.

**Figure 7:**
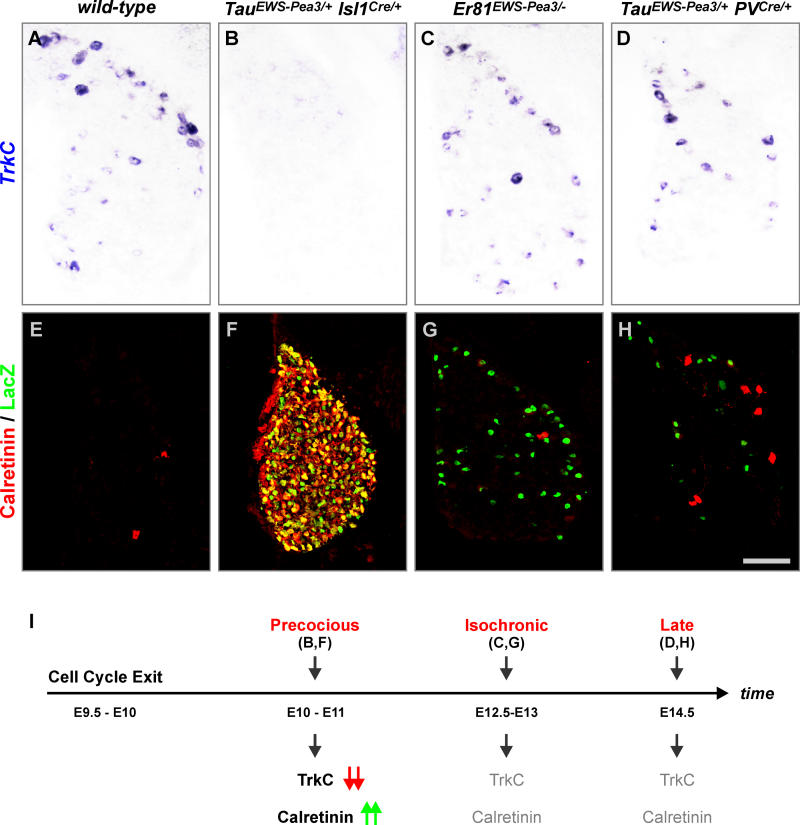
Gene Expression Analysis upon Induction of Precocious or Isochronic ETS Signaling (A–H) Analysis of *TrkC* expression by in situ hybridization (A–D), or Calretinin (red) and LacZ (green) expression by immunohistochemistry (E–H), on E16.5 lumbar DRG of wild-type (A and E), *Tau^EWS-Pea3/+^ Isl1^Cre/+^* (B and F), *Er81^EWS-Pea3/−^* (C and G), and *Tau^EWS-Pea3/+^ PV^Cre/+^* (D and H) embryos. (I) Summary diagram illustrating deregulation of TrkC (red arrows, downregulation) and Calretinin (green arrows, upregulation) expression upon precocious (B and F) induction of EWS-Pea3 expression in DRG neurons (B and F; E10–E11, i.e., shortly after cell cycle exit, E9.5–E10). In contrast, activation of EWS-Pea3 from the endogenous *Er81* locus (C and G; E12.5–E13) or via Cre recombinase expression from the *PV* locus activating late expression from the *Tau* locus (D and H; E14.5) does not interfere with the normal expression of *TrkC* and Calretinin (shown in grey). Scale bar: (A–D), 65 μm; (E–H), 80 μm.

**Figure 8:**
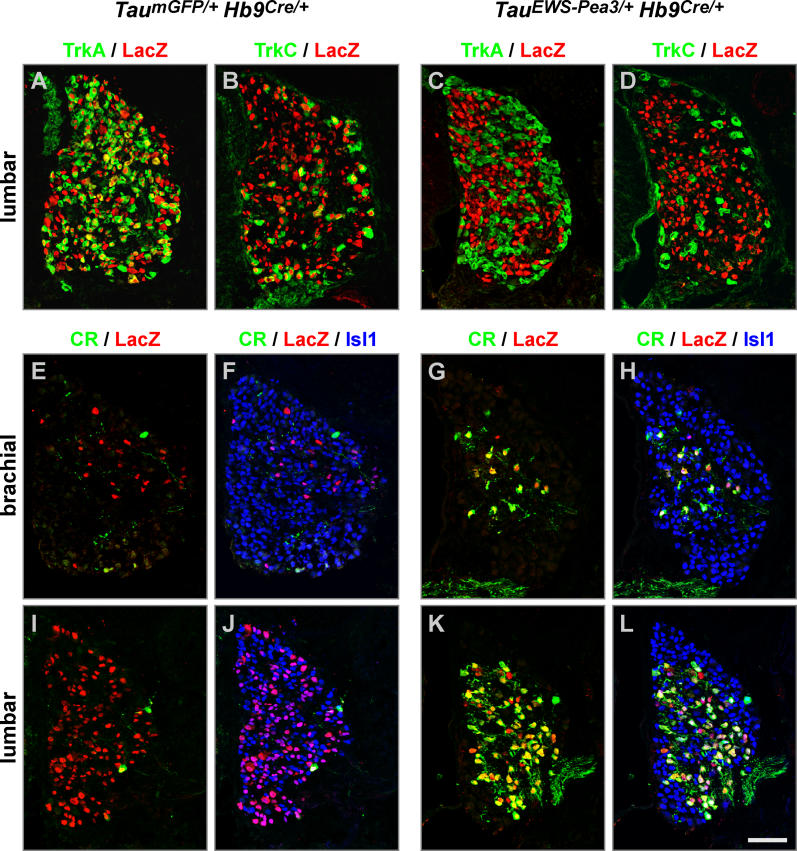
Precocious ETS Signaling Induces Gene Expression Changes Cell-Autonomously (A–D) Expression of TrkA (A and C; green) or TrkC (B and D; green), and LacZ (red), in E16.5 lumbar DRG of *Tau^mGFP/+^ Hb9^Cre/+^* (A and B) and *Tau^EWS-Pea3/+^ Hb9^Cre/+^* (C and D) embryos. (E–L) Expression of Calretinin (green), LacZ (red), and Isl1 (F, J, H, and L; blue) in E16.5 brachial (E–H) and lumbar (I–L) DRG of *Tau^mGFP/+^ Hb9^Cre/+^* (E, F, I, and J) and *Tau^EWS-Pea3/+^ Hb9^Cre/+^* (G, H, K, and L) embryos. Scale bar: (A–D), 80 μm; (E–L), 70 μm.

**Figure 9:**
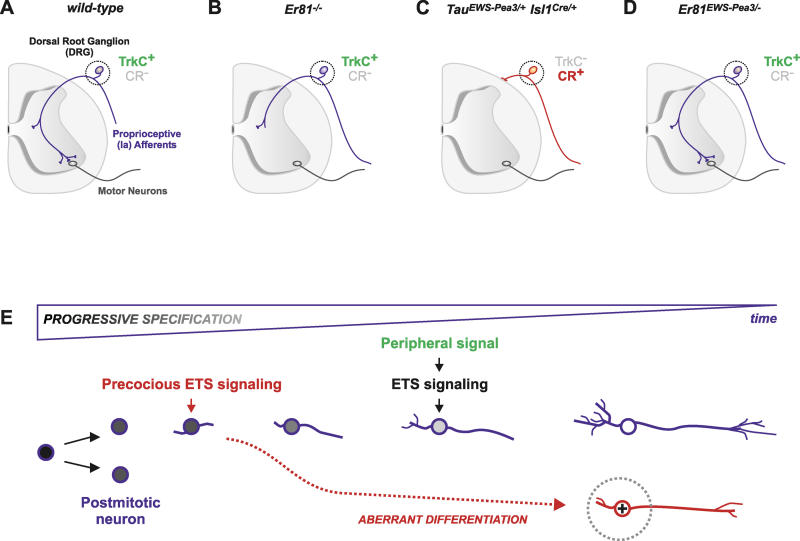
Progressive Neuronal Specification Is Paralleled by a Developmental Shift in Response to ETS Transcription Factor Signaling Schematic summary diagram illustrating the importance of temporally appropriate upregulation of transcription factor expression during specification of DRG neurons for late aspects of neuronal differentiation and circuit assembly. (A–D) Expression of *EWS-Pea3* from the endogenous *Er81* locus can rescue anatomical defects observed in *Er81^−/−^* mice, and no change in expression of TrkC (green) or Calretinin (CR; grey) is observed in proprioceptive afferents (A, B, and D). In contrast, precocious ETS signaling leads to severe defects in the establishment of DRG neuronal projections accompanied by inappropriate gene expression changes (C; upregulation of CR (red) and downregulation of TrkC [grey]). (E) Precocious ETS signaling (red) during progressive specification of proprioceptive sensory neurons leads to aberrant neuronal differentiation (red dashed line). In contrast, the isochronic, target-induced (green; peripheral signal) onset of ETS transcription factor signaling (black) induces appropriate terminal neuronal differentiation (blue).

We first analyzed expression of *TrkC,* a gene downregulated in DRG neurons of *Tau^EWS-Pea3/+^ Isl1^Cre/+^* embryos (Figure 7A and 7B). The level of expression of *TrkC* was indistinguishable from wild-type in DRG neurons of *Er81^EWS-Pea3/−^* and *Tau^EWS-Pea3/+^ PV^Cre/+^* embryos (Figure 7A, 7C, and 7D). Moreover, PV was not expressed in DRG neurons of *Tau^EWS-Pea3/+^ Isl1^Cre/+^* embryos (Figure S5) but was expressed by proprioceptive afferents in both wild-type and *Er81^EWS-Pea3/−^* embryos (see Figures 1 and S5) [14]. We also found several genes that were ectopically upregulated in DRG neurons of *Tau^EWS-Pea3/+^ Isl1^Cre/+^* embryos (Figure 7). Calretinin and Calbindin, two different calcium-binding proteins expressed by subpopulations of DRG neurons in wild-type, *Er81^EWS-Pea3/−^,* and *Tau^EWS-Pea3/+^ PV^Cre/+^* embryos (Figure 7E, 7G, and 7H; data not shown) [31,32], were induced in more than 95% of all DRG neurons of *Tau^EWS-Pea3/+^ Isl1^Cre/+^* embryos (Figures 7F and S5). These findings suggest that DRG neurons in *Tau^EWS-Pea3/+^ Isl1^Cre/+^* embryos fail to differentiate to a normal fate and instead acquire an aberrant identity distinct from any subpopulation of wild-type DRG neurons. Finally, to assess whether EWS-Pea3 expressed precociously acts exclusively cell-autonomously or whether it may also influence neighboring DRG neurons, we activated expression of EWS-Pea3 using *Hb9^Cre^* mice [33]. Due to a transient and rostro-caudally graded expression of *Hb9* at neural plate stages, very few DRG neurons at brachial levels and increasingly more neurons progressing caudally undergo recombination in *Tau^EWS-Pea3/+^ Hb9^Cre/+^* and *Tau^mGFP/+^ Hb9^Cre/+^* embryos (Figure 8). Nevertheless, downregulation of Trk receptor expression or upregulation of Calretinin is restricted exclusively to neurons that have undergone recombination and cannot be observed in *Tau^mGFP/+^ Hb9^Cre/+^* embryos (Figure 8). Together, these results and the findings obtained from in vitro culture experiments (see Figures 4 and 5) demonstrate that precocious or isochronic expression of *EWS-Pea3* in the same neurons leads to significantly different cell-autonomous cellular responses with respect to gene expression, neuronal survival, and neurite outgrowth (Figure 9).

## Discussion

Target-derived signals exhibit a conserved role in the induction of defined programs of transcription factor expression late in post-mitotic neuronal differentiation [10]. This study provides evidence that the late onset of transcription factor expression is essential for many later aspects of neuronal differentiation and circuit formation. Our data indicate that DRG neurons undergo a temporal change in their competence to respond to ETS transcription factor signaling, as assessed by changes in gene expression and axonal target invasion (Figure 9). Our findings argue for the necessity of target-induced, and therefore temporally controlled, upregulation of ETS transcription factor signaling. More generally, they suggest that temporally regulated activation of transcriptional programs coupled to a particular fate induced in neurons at early developmental stages represents an important mechanism of neuronal maturation.

One striking observation of this study is that precocious induction of ETS signaling promotes neuronal survival without a requirement for neurotrophic support and in complete absence of Trk receptor expression. In contrast, ETS signaling at the normal time of onset of *Er81* expression does not result in enhanced neuronal survival in the absence of neurotrophins and also does not lead to downregulation of TrkC expression in proprioceptive afferents. These findings demonstrate very distinct activities of one transcriptional regulator at different developmental steps within a committed post-mitotic neuronal lineage. The absence of Trk receptor expression upon precocious induction of ETS signaling can only partially explain the observed phenotype in axonal projections. Elimination of TrkA receptor signaling in *Bax* mutant mice perturbs establishment of peripheral projections of cutaneous afferents, whereas establishment of central projections does not appear to be affected [8]. In the absence of NT-3 signaling, development of central as well as peripheral proprioceptive afferent projections is perturbed [9]. In contrast, upon precocious induction of ETS signaling, we found more pronounced defects in the establishment of central rather than peripheral projections for all DRG neurons.

Induction of Er81 expression in proprioceptive afferents is controlled by peripheral NT-3 as axons reach the vicinity of target muscles, and thus occurs only approximately 3 d after proprioceptive neurons become post-mitotic [9,14]. This temporally delayed and target-induced upregulation of ETS transcription factor expression several days after a neuronal lineage of a specific identity first emerges is not restricted to DRG sensory neurons, but is also found in motor neuron pools [13]. Target-derived factors have also been implicated in controlling neuronal maturation of predetermined neurons in *Drosophila,* in which expression of members of the BMP family in the target region is essential for the induction of mature peptidergic properties in a subpopulation of neurons marked by the coordinate expression of the two transcription factors Apterous and Squeeze [11,12]. Thus, both in *Drosophila* and vertebrates, target-derived factors appear to act permissively to induce the expression of transcriptional programs involved in terminal neuronal maturation.

Our findings are compatible with a model in which DRG neurons acquire their mature fate by sequential and temporally controlled addition of lineage-specific features (Figure 9). Target-derived factors act on predetermined neuronal lineages to switch their developmental programs to become compatible with processes such as target invasion and branching. Such a transition state in the acquisition of a defined neuronal fate would be accompanied by the induction of appropriate transcriptional programs through the expression of specific transcription factors. Mechanisms such as chromosomal remodeling that restrict or expand access to certain target genes [34] or activation by cofactors responsible for changing the action of particular transcription factors [35] could represent possible mechanisms by which the downstream transcriptional profile of a transcription factor could be temporally shifted towards the selection and control of distinct target genes. Interestingly, several ETS transcription factors are activated through release of autoinhibition via interaction with cofactors and/or via post-translational modifications [35,36,37]. The fusion of EWS with Pea3 could circumvent a need for activation through specific cofactors while still maintaining ETS site dependence, thus rendering EWS-Pea3 less sensitive to the cellular context than endogenous ETS transcription factors. Using this fusion protein, our experiments demonstrate a profound change in the action of ETS signaling at the level of transcriptional regulation within post-mitotic DRG neurons over time. Moreover, the observed transcriptional shift in ETS signaling is paired with the onset of appropriate regulation of neuronal subtype specification and establishment of axonal projections into the target area.

Recent experiments addressing the temporal constraints of transcription factor action in proliferating neural progenitor cells adds to the idea that defined temporal windows, during which transcription factors act to control distinct downstream target genes, are of key importance to neuronal fate acquisition. During *Drosophila* neuroblast generation, the transcription factor Hunchback controls specification and differentiation of early-born neuroblasts [38]. Over time, however, neuroblasts progressively lose their competence to generate cells of an early fate in response to Hunchback expression [39]. These findings thus also argue for a change in cellular competence to respond to a specific transcription factor over time albeit in an early precursor context. More generally, during the differentiation of hematopoietic lineages, several transcription factors have also been shown to exhibit distinct functions at progressive steps of lineage specification [40]. Analysis of the mechanisms by which transcription factor programs can be shifted over time to control different complements of downstream genes and thus different aspects of neuronal and cellular fates in progenitor cells or post-mitotic neurons may provide further insight into the way in which transcription factors act to control the assembly of neuronal circuits.

## Materials and Methods

### 

#### Generation of transgenic mice and mouse genetics

*Er81^EWS-Pea3^* mice were generated following a strategy similar to that described for the generation of *Er81^NLZ^* mice [14]. In brief, a targeting vector with a cDNA coding for *EWS-Pea3* was inserted in frame with the endogenous start ATG into exon 2 of the *Er81* genomic locus and used for homologous recombination in W95 ES cells. *EWS-Pea3* represents a fusion gene between the amino terminal of EWS and the ETS domain of Pea3 [20]. The primer pair used to specifically detect the *Er81^EWS-Pea3^* allele was 5′- CAGCCACTGCACCTACAAGAC-3′ and 5′- CTTCCTGCTTGATGTCTCCTTC-3′. For the generation of *Tau^mGFP^* and *Tau^EWS-Pea3^* mice, *lox-STOP-lox-mGFP-IRES-NLS-LacZ-pA* and *lox-STOP-lox-EWS-Pea3-IRES-NLS-LacZ-pA* targeting cassettes were integrated into exon 2 of the *Tau* genomic locus (the endogenous start ATG was removed in the targeting vectors; details available upon request). mGFP was provided by P. Caroni [25]. ES cell recombinants were screened by Southern blot analysis using the probe in the 5′ region as described previously [41]. Frequency of recombination in 129/Ola ES cells was approximately 1/3 for both *Tau* constructs. For the generation of *PV^Cre^* mice, mouse genomic clones were obtained by screening a 129SV/J genomic library (Incyte, Wilmington, Delaware, United States). For details on the genomic structure of the mouse *PV* locus see [42]. An *IRES-Cre-pA* targeting cassette [33] was integrated into the 3′ UTR of exon 5, and ES cell recombinants were screened with a 5′ probe (oligos, 5′- GAGATGACCCAGCCAGGATGCCTC-3′ and 5′- CTGACCACTCTCGCTCCGGTGTCC-3′; genomic DNA, HindIII digest). The frequency of recombination in 129/Ola ES cells was approximately 1/20. Recombinant clones were aggregated with morula stage embryos to generate chimeric founder mice that transmitted the mutant alleles. In all experiments performed in this study, animals were of mixed genetic background (129/Ola and C57Bl6). *Thy1^spGFP^* transgenic mice were generated in analogy to De Paola et al. [25], and for these experiments a strain of mice with early embryonic expression was selected. *Isl1^Cre^* and *Hb9^Cre^* mouse strains have been described [33,43] and *Bax^+/−^* animals were from Jackson Laboratory (Bar Harbor, Maine, United States) [27]. Timed pregnancies were set up to generate embryos of different developmental stages with all genotypes described throughout the study.

#### Transcriptional transactivation assays

The following plasmids were used for transcriptional transactivation assays: pRc/RSV (Invitrogen, Carlsbad, California, United States), pRc/RSV-Er81, pRc/RSV-EWS-Pea3, pTP-5xETS, and pTP-5xETSmut. pRc/RSV-Er81 and pRc/RSV-EWS-Pea3 were obtained by insertion of the cDNAs for Er81 or EWS-Pea3 (gift from J. A. Hassell) into pRc/RSV. pTP-5xETS was constructed by inserting a cassette of five repetitive copies of high-affinity Pea3 binding sites (5′- GCCGGAAGC-3′) [18,19] into a modified version of pTK-Luc. pTP-5xETSmut was generated as pTP-5xETS but using a mutated complement of the Pea3 binding sites (5′- GCCTATGGC-3′). A control plasmid to normalize for transfection efficiency (placZ) and pTK-Luc were a gift from D. Kressler. COS-7 cells were co-transfected with 1–1.2 μg of total DNA including one of the effector plasmids pRc/RSV-empty, pRc/RSV-Er81, or pRc/RSV-EWS-Pea3; one of the reporter plasmids pTP-5xETS or pTP-5xETSmut; and placZ. Cells were harvested after 25 h and processed for assays to determine luciferase and LacZ activity as described previously [44]. Luciferase values normalized to LacZ activity are referred to as luciferase units.

#### In situ hybridization and immunohistochemistry

For in situ hybridization analysis, cryostat sections were hybridized using digoxigenin-labeled probes [45] directed against mouse *TrkA* or *TrkB,* or rat *TrkC* (gift from L. F. Parada). Antibodies used in this study were as follows: rabbit anti-Er81 [14], rabbit anti-Pea3 [14], rabbit anti-PV [14], rabbit anti-eGFP (Molecular Probes, Eugene, Oregon, United States), rabbit anti-Calbindin, rabbit anti-Calretinin (Swant, Bellinzona, Switzerland), rabbit anti-CGRP (Chemicon, Temecula, California, United States), rabbit anti-vGlut1 (Synaptic Systems, Goettingen, Germany), rabbit anti-Brn3a (gift from E. Turner), rabbit anti-TrkA and -p75 (gift from L. F. Reichardt), rabbit anti-Runx3 (Kramer and Arber, unpublished reagent), rabbit anti-Rhodamine (Molecular Probes), mouse anti-neurofilament (American Type Culture Collection, Manassas, Virginia, United States), sheep anti-eGFP (Biogenesis, Poole, United Kingdom), goat anti-LacZ [14], goat anti-TrkC (gift from L. F. Reichardt), and guinea pig anti-Isl1 [14]. Terminal deoxynucleotidyl transferase-mediated biotinylated UTP nick end labeling (TUNEL) to detect apoptotic cells in E13.5 DRG on cryostat sections was performed as described by the manufacturer (Roche, Basel, Switzerland). Quantitative analysis of TUNEL^+^ DRG cells was performed essentially as described [27]. BrdU pulse-chase experiments and LacZ wholemount stainings were performed as previously described [46]. For anterograde tracing experiments to visualize projections of sensory neurons, rhodamine-conjugated dextran (Molecular Probes) was injected into single lumbar (L3) DRG at E13.5 or applied to whole lumbar dorsal roots (L3) at postnatal day (P) 5 using glass capillaries. After injection, animals were incubated for 2–3 h (E13.5) or overnight (P5). Cryostat sections were processed for immunohistochemistry as described [14] using fluorophore-conjugated secondary antibodies (1:1,000, Molecular Probes). Images were collected on an Olympus (Tokyo, Japan) confocal microscope. Images from in situ hybridization experiments were collected with an RT-SPOT camera (Diagnostic Instruments, Sterling Heights, Michigan, United States), and Corel (Eden Prairie, Minnesota, United States) Photo Paint 10.0 was used for digital processing of images.

#### In vitro cultures of DRG

Individual lumbar DRG were dissected from E13.5 or E14.5 embryos and placed on Matrigel (BD Biosciences, San Jose, California, United States) coated coverslips in DMEM/F12 (Gibco, San Diego, California, United States), 2 mM L-Gln (Gibco), N2 (Gibco), and 1 mg/ml BSA (Sigma, St. Louis, Missouri, United States) without neurotrophins, or supplemented with either NGF (100 ng/ml, Gibco) or NT-3 (20 ng/ml, Sigma). DRG explants (*n* ≥ 20 for each condition) were cultured for 48 h, processed for immunocytochemistry, and analyzed using confocal microscopy.

#### Western blot analysis

Lumbar DRG from E16.5 embryos were isolated, mechanically disrupted, homogenized using glass beads (Sigma), and lysed in standard lysis buffer supplemented with protease and phosphatase inhibitors as described [47]. Protein extracts were resolved by SDS-PAGE, and immunoblotting was performed using antibodies against Akt, p-Akt (Ser473), CREB, p-CREB (Ser133), Bax, Bcl-xl (Cell Signaling Technology, Beverly, Massachusetts, United States), and Bcl2 (BD Pharmingen, San Diego, California, United States). For quantification, films (X-OMAT AR, Eastman Kodak, Rochester, New York, United States) were scanned and densitometry was performed using IMAGEQUANT 5.2 (Molecular Dynamics, Amersham, Uppsala, Sweden).

#### Electrophysiology

Electrophysiological analysis was performed as previously described [48]. Briefly, intracellular recordings from identified quadriceps motor neurons were made using sharp electrodes (75–120 MΩ, 3M KCl). Average responses (10–20 trials) from suprathreshold nerve stimulation (1.5 times the strength that evokes maximal monosynaptic response) of the quadriceps nerve were acquired with LTP software [49]. Only cells with stable resting potentials more negative than −50 mV were considered for analysis. Monosynaptic amplitudes were determined offline using custom routines in the Matlab environment (The Mathworks, Natick, Massachusetts, United States) as previously described [48].

## Supporting Information

Figure S1 Gene Expression Analysis in DRG Neurons of *Er81^EWS-Pea3^* Mice Analysis of *TrkC* expression by in situ hybridization (A and E), and Runx3 (red; B and F), Brn3A (red; C and G), Isl1 (red; D and H), p75 (red; I and M), TrkA (red; J and N), CGRP (red; K and O), Calretinin (CR; red; L and P), and LacZ (green; B–D and F–P) by immunohistochemistry on E16.5 lumbar DRG of *Er81^NLZ/+^* (A–D and I–L) and *Er81^EWS-Pea3/−^* (E–H and M–P) embryos.
Scale bar: (A, B, E, F, L, and P), 70 μm; (C, D, G, H, I–K, and M–O), 75 μm.
(4.9 MB CDR).

Figure S2 Ia Proprioceptive Afferents Make Functional Connections with Motor Neurons in *Er81^EWS-Pea3^* Mutants (A and B) Representative traces from intracellular recordings measuring Ia afferent monosynaptic input to quadriceps motor neurons evoked by suprathreshold stimulation of the quadriceps nerve in wild-type (A) and *Er81^EWS-Pea3/−^* mutant (B) animals.
(C) Average monosynaptic amplitudes (± standard error of the mean) from all recorded cells (wild-type, *n* = 11; mutant, *n* = 8).
(20 KB CDR).

Figure S3 Generation of Mice Expressing *EWS-Pea3* or *mGFP* in Early Post-Mitotic DRG Neurons (A) Top panel shows organization of the *Tau* genomic locus in the region targeted by homologous recombination in analogy to Tucker et al. [41]. Exons 1–3 are shown as light blue boxes, and the *Tau* start codon in exon 2 is indicated as ATG. The probe used to detect homologous recombination is shown as a grey box. Middle and bottom panels show *Tau* locus after homologous recombination to integrate targeting cassettes (green) into exon 2 with coincident elimination the endogenous *Tau* start codon. The integrated targeting cassettes allow for conditional expression of *EWS-Pea3* and *NLS-LacZ (NLZ)* (middle) or *mGFP* and *NLS-LacZ* (bottom) upon Cre-recombinase-mediated activation. In the absence of Cre recombinase, a transcriptional stop sequence flanked by *loxP* sites inhibits expression of the respective transgenes from their start codons (ATG in grey).
(B) Southern blot analysis of *Tau^EWS-Pea3/+^* and *Tau^mGFP/+^* genomic DNA to detect the mutant allele.
(C) In the presence of Cre recombinase, the transcriptional stop sequence in the cassettes integrated into the *Tau* locus is removed. Expression of *EWS-Pea3* and *NLS-LacZ* (top) or *mGFP* and *NLS-LacZ* (bottom) can now occur in neurons coincidently expressing Cre recombinase and *Tau* (indicated as ATG in green).
(D–L) Expression of Isl1 (D, G, and J), EWS-Pea3 (E and H), GFP (K), or LacZ (F, I, and L), in E12 (D–I) or E13.5 (J–L) DRG neurons of wild-type (D–F), *Tau^EWS-Pea3/+^ Isl1^Cre/+^* (G–I), and *Tau^mGFP/+^ Isl1^Cre/+^* (J–L) embryos.
Scale bar: (D–F), 40 μm; (G–I), 35 μm; (J–K), 50 μm.
(1.5 MB CDR).

Figure S4 Generation of *PV^Cre^* Mice (A) Above is the organization of the *PV* genomic locus. Exons are schematically illustrated as light blue boxes, where exon 2 contains the start codon (ATG) and exon 5 contains the stop codon (STOP). Probe to screen for homologous recombination is shown as grey box. Below is a schematic diagram to show the *PV* locus after the integration of an *IRES-Cre* cassette (green) 3′ to the translational stop codon of *PV* using homologous recombination in ES cells.
(B) Southern blot analysis of *PV^Cre^* wild-type (*+/+*) and heterozygous (*+/−*) genomic DNA using the probe indicated in (A).
(C and D) Expression of GFP (green) and LacZ (red; C) or PV (red; D) in P0 *Tau^mGFP/+^ PV^Cre/+^* mice. Note that more than 90% of PV^+^ neurons coexpress GFP (D; data not shown).
Scale bar: (C and D), 50 μm.
(2 MB CDR).

Figure S5 Gene Expression Analysis upon Precocious Induction of EWS-Pea3 in DRG Neurons Immunohistochemical analysis of PV (A and D), Calretinin (B and E), and Calbindin (C and F) expression on E16.5 lumbar DRG of wild-type (A–C) and *Tau^EWS-Pea3/+^ Isl1^Cre/+^* (D–F) embryos.
Scale bar: 80 μm.
(950 KB CDR).

## Abbreviations

DRG: dorsal root ganglion/ganglia
E: embryonic day
EWS: Ewing sarcoma
mGFP: membrane-targeted green fluorescent protein
NT-3: neurotrophin 3
P: postnatal day
PV: Parvalbumin
spGFP: synaptophysin green fluorescent protein
